# External Cause Coding of Injury Encounters in the Military Health System Among Active Component U.S. Service Members, 2016–2019

**Published:** 2025-02-20

**Authors:** Michelle Canham-Chervak, Anna Schuh-Renner, Shauna L. Stahlman, Catherine Rappole, Bruce H. Jones

**Affiliations:** 1 Injury Prevention Branch, Defense Health Agency Public Health–Aberdeen, MD; 2 Epidemiology and Analysis Branch, Armed Forces Health Surveillance Division, Public Health Directorate, Defense Health Agency, Silver Spring, MD; 3 Clinical Public Health and Epidemiology Directorate, Defense Centers for Public Health–Aberdeen

## Abstract

Knowledge of injury causes is essential for prevention. To investigate cause coding in service members’ electronic medical records, injury encounters from 2016 to 2019 containing at least 1 external cause code were analyzed. Approximately 10% of incident injury encounters contained at least 1 cause code describing the mechanism, activity, or place of occurrence. Less than 2% of overuse injury encounters had a cause code each year, compared to 36.4–44.0% of acute injuries occurring from 2016 to 2019. Cause coding occurred more frequently in records from military facilities compared to outsourced care (p<0.001). Inpatient records were more likely to be cause-coded than outpatient records (p<0.001). More injury encounters in emergency clinics were cause coded (>50%), compared to approximately 7% of primary care and 2% of specialist encounters. In 2019, the leading mechanism was overexertion (19.9%), followed by falls, slips, or trips (18.7%). The primary activity associated with injuries was running (21.1%). Military training ground was the leading place of occurrence (13.0%). Improvements to the quality and quantity of external cause coding in the medical records would provide critical details to inform military injury prevention.

**What are the new findings?:**

From 2016 through 2019, approximately 10% of 1.5 million annual U.S. service member incident injury medical encounters contained external cause codes. Acute injuries were approximately 20 times more likely to receive a cause code than overuse injuries. Causes were less likely to be recorded in outpatient care records and at non-military health care facilities.

**What is the impact on readiness and force health protection?:**

Injuries are the leading reason for service members to seek health care and contribute significantly to military medical non-readiness. Specific and accurate recording of injury cause codes by health care providers establishes and develops a data-informed mechanism for the design, implementation, prioritization, and monitoring of interventions and prevention programs to reduce injury risk among service members.

## 1 BACKGROUND

Injuries have been the leading reason for medical encounters and limited duty among U.S. active duty service members.^1^ In 2018, 2 of every 5 medical encounters among service members were due to injury, resulting in over 4.7 million encounters affecting over 675,000 service members.^2^ Military injury surveillance efforts have estimated that injuries annually result in approximately 25 million days of limited duty within the U.S. Army, Navy, Marine Corps, and Air Force.^3^

U.S. service members receive care for injuries and other health conditions through the Military Health System (MHS), which has a dual health care and readiness mission with a focus on promoting and sustaining health.^4,5^ As part of a health care encounter, diagnosis and cause codes are entered into electronic medical records by health care providers and, when possible, by medical coders for selected care such as hospitalizations or emergency clinic visits. External cause of injury codes can capture the intent (unintentional or intentional), how the injury occurred (mechanism), the activity at the time of the injury event (activity), and the location where the event occurred (place of occurrence). For injuries, summaries of cause codes from electronic medical records facilitate a data-driven approach and optimize resources by directing efforts to develop relevant injury prevention and treatment plans.^6,7^

There is no national requirement to assign external cause of injury codes in medical records,^8^ although the value of injury cause coding to identify intervention opportunities and monitor effects of prevention programs and policies has been noted in International Classification of Diseases, 10th Revision, Clinical Modification (ICD-10-CM) coding guidance and previous epidemiological investigations.^8,9,10,11^ Military leaders recognize that cause information is needed to reduce injuries.^12,13,14^ To date, however, injury cause coding of military medical records remains incomplete.^9,10,15,16^

Previous publications have summarized external causes of injury for subsets of U.S. military data.^10,16,17,18^ The purpose of this article is to describe causes of injury for all U.S. service members, from 2016 through 2019, and identify variations in injury cause coding over time and by branch of military service, type of health care visit and facility, and diagnosis category.

## 2 METHODS

Data consisted of injury medical records maintained in the Defense Medical Surveillance System that were obtained by the authors from the Armed Forces Health Surveillance Division in 2022. Specifically, records were obtained from the Comprehensive Ambulatory/Professional Encounter Record (CAPER), Standard Inpatient Data Record (SIDR), and TRICARE Encounter Data Non-Institutional (TED-NI) and Institutional (TED-I) files. Prior surveillance analyses indicated that more than 99.5% of incident injury records contained 9 diagnoses or less, therefore 9 diagnosis (DX) positions were requested. The records documented ambulatory (outpatient) encounters and hospitalizations (inpatient) that occurred in fixed military medical facilities worldwide and civilian treatment facilities (outsourced care) if reimbursement was sought through the MHS.

The Taxonomy of Injuries^19^ was used to identify injuries, from 2016 through 2019, among active component service members in the Army, Navy, Marine Corps, or Air Force. Diagnoses are primarily from ICD-10-CM Chapter 13 (‘M’ codes primarily for micro-traumatic overuse injuries; diseases of the musculoskeletal system and connective tissue) and Chapter 19 (‘S’ and ‘T’ codes for acute injuries; injury, poisoning, and certain other consequences of external causes). Incident injury diagnoses in the primary diagnosis (DX1) position matching Taxonomy diagnosis codes were included, in accordance with standardized military injury surveillance methodology, excluding codes for subsequent and sequela encounters (i.e., ICD-10-CM codes with D or S suffixes). Incident injuries were the focus of the analysis, given that MHS coding guidance specifies assignment of external cause codes to initial encounters. To identify incident injuries, a 60-day gap-in-care rule was applied by injury type and injured body part, to exclude follow-up visits for the same service members within 60 days.^19^

Next, injury medical records containing at least 1 external cause code in diagnosis (DX) positions (1-9) were identified. National Center for Health Statistics (NCHS) categorizations of external cause codes (ICD-10-CM Chapter 20, ‘V’-‘Y’ codes) were adapted for use.^20^ In alignment with NCHS, a subset of injury diagnosis codes from ICD-10-CM Chapter 19 that describe the injury mechanism were also included as cause codes (T14.91, T15-T19, T36-T65, T71, T73-T76, U07, V00-V99, W00-X58, X71-X83, X92-Y09, Y21-Y33, Y35-Y38). Cause codes of all intentions (unintentional, intentional, assault, legal intervention/war, undetermined) were included. Codes for unspecified mechanisms were identified in accordance with the NCHS-defined ‘Unspecified’ category.^20^ Given that these codes do not provide actionable information for injury prevention, records that included only these unspecified codes were excluded, but counts are noted in table footnotes. If an unspecified cause code was used in conjunction with a more detailed cause code, the more detailed cause was reported.

Activity codes are ICD-10-CM external cause codes with ‘Y93’ as the first 3 digits in any diagnosis position.^8,21^ Similarly, place of occurrence codes are any cause codes with ‘Y92’ as the first 3 digits in any diagnosis position.^8,21^ Activity and place of occurrence subsets each have only 1 Unspecified code, Y93.9 (“Activity, unspecified”) and Y92.9 (“Unspecified place or not applicable”), which were excluded from this analysis in a similar fashion as the unspecified mechanism codes.

The percentage of incident injury records with at least 1 external cause code are reported by ICD-10-CM chapter, care source (direct or outsourced), visit type (inpatient or outpatient), military treatment facility type (medical center, hospital, or clinic),^22^ and clinic type (emergency, primary care, specialist). Military treatment facility type for each record was identified by the Defense Medical Information System identifier assigned to the record. For outpatient military treatment facility encounters, Medical Expense and Performance Reporting System codes^23^ were also provided and used for categorization into 3 broad clinical groups: emergency, primary care, and specialist.

Data prior to 2020 are presented in this report, due to the fact that more recent data were affected by pandemic-related changes in service member health care provision and the transition to a new electronic health record, MHS GENESIS.^24^ Data were not available for 4 sites—Naval Health Clinic Oak Harbor, Naval Hospital Bremerton, Air Force Medical Services Fairchild, Madigan Army Medical Center—that were the first to transition to GENESIS from 2017 through 2019; these sites were not included in this analysis, due to data completeness concerns related to this initial transition period.

Statistical analyses were conducted in SAS™ version 9.4. Proportions of incident injuries receiving mechanism, activity, or place codes are reported. Chi-square tests were used to evaluate differences in proportions across categories and identify statistically significant temporal trends. This project was reviewed and approved as public health practice by the Defense Centers for Public Health–Aberdeen (DCPH-A) Public Health Review Board.

## 3 RESULTS

From 2016 through 2019, there were 5,973,994 incident medical encounter records for injuries across all services. Only 10.0% of incident injury encounter records (n=594,404) received a cause code (**Table 1**). **Tables 1** and **2** show the numbers and percentages of cause-coded incident injuries.

On average, there were 118,000 total mechanism cause codes assigned to injury records each year (range: 101,281-131,105), including instances in which multiple codes were assigned to the same injury (**Table 1**). During this period, on average, 7.9% of incident injury records were given a mechanism code, 3.8% received an activity code, and 2.5% received a place of occurrence code annually.

From 2016 through 2019, 9–16% of mechanism cause codes were categorized as Unspecified (e.g., X58.X, “exposure to other specified factors”; Y37.90, “military operations, unspecified”). Likewise, 3–6% of activity codes and 17–22% of place of occurrence codes were Unspecified codes. Unspecified codes are reported in the footnotes of **Tables 1** and **2**.

Among all services, there were more incident injury records with at least 1 cause code in later years, increasing from 9.2% in 2016 to 10.3% in 2019 (**Table 1**, p<0.0001 for all comparisons). Compared to other services, the Army had a higher proportion (range: 10.0-11.7%) of records with at least 1 cause code (p<0.001).

Incident injury records with ‘S’ and ‘T’ diagnosis codes (predominantly acute injuries) contained at least 1 cause code over one-third of the time (**Table 2**) and around 20 times more often than injuries receiving an ‘M’ code (overuse injuries, <2% cause-coded) or other diagnoses (about 1%) (p<0.001). A comparison by care source (**Table 2**) shows a significantly higher proportion of cause-coded incident injury records at military hospitals and clinics (range: 9.5-10.8%) compared to outsourced care facilities (range: 7.7-8.2%). In addition, inpatient injury records (range: 32.0-40.5%) were more likely to have a cause code compared to outpatient care (range: 9.2-10.3%). Considering treatment facility size, for all services incident injuries treated at military hospitals had the highest proportions of cause-coded records (range: 17.5-19.6%), followed by military medical centers (range: 17.0-19.5%) and clinics (range: 6.1-7.1%). From 2016 through 2019, more than half (range: 53.1-57.1%) of emergency care injury records at military facilities were cause-coded, compared to around 7% from primary care (range: 6.3-7.4%) and less than 3% from specialty care (range: 2.3-2.9%).

After the ICD-10-CM ‘Overexertion’ mechanism cause code was introduced in 2017,^17^ the proportion of injury records cause-coded as Overexertion increased significantly (plgt;0.001), from 16.4% that year to 19.9% in 2019 (**Table 3**). Overexertion was the leading mechanism of injury in 2018 and 2019. Other frequently coded mechanisms of injury during the 4-year period included “falls/slips/trips” (range: 18.3-23.1%) and “struck by/against” (range: 17.4-21.6%).

Among external cause codes related to activity (**Table 4**), the most frequently coded activity associated with injury was running (approximately 20% each year), followed by “Other specified” and “Walking/marching/hiking.” The proportion of injuries with activity codes for Walking/marching/hiking increased steadily in the 4-year period, from 7.7% in 2016 to 10.6% in 2019. Frequently coded places of occurrence for injuries (**Table 4**) were “Military training ground,” “Other specified places,” “Unspecified places in private residences,” and “Other specified sports and athletic areas.”

For all external cause codes, use of ‘Other specified’ codes for mechanism^20^ (e.g., Other specified child/adult abuse, Other specified foreign body) as well as activity (Y93.83) and place of occurrence (Y92.89) were lower in 2019 compared to 2016 (p<0.001).

## 4 DISCUSSION

This is the first comprehensive assessment of ICD-10-CM external cause coding of military electronic injury medical records. Overall, the proportion of injury records with cause coding is small and substantially less than historical military injury hospitalization cause coding rates.^25^ More frequent and more specific cause coding is needed in outpatient settings, where the majority (99%) of injury treatment occurs.^9^

Cause coding was more common with acute injuries (S and T codes), compared to overuse injuries (M codes). This is not surprising, given that national injury categorization tools focus on acute injuries only.^21,26^ Approximately 75% of service member injuries are due to cumulative microtrauma, however, and such injuries are routinely included in injury definitions by sports and occupational medicine experts.^27^ These overuse injuries, which range from joint pain to Achilles tendonitis and stress fractures, are common in physically active populations and result from often preventable factors such as over-training, over-exertion, repetitive movement, vibration, and prolonged static postures.^27^ To effectively address military injuries, cause information is needed for both acute and overuse injuries.

Cause coding was also shown to be more common at military treatment facilities, especially hospitals and medical centers. This may be because larger facilities have resources including medical coders who train providers and audit and code records. Emergency care departments, on average, cause coded a much higher proportion of injury records compared to other clinics, with roughly half of injury-related emergency department injury records receiving a cause code. This was consistent across services, suggesting that processes and staffing in emergency care facilitated cause coding.

While assignment of external cause codes is not mandatory in the U.S. or the MHS, annual ICD-10-CM coding guidelines consistently recommend that providers voluntarily report external cause, given its value for injury research and evaluation of prevention strategies.^8^ In addition, the military safety community has recognized the need for injury cause coding to support the systematic identification and mitigation of Department of Defense (DOD) injuries.^3,13^ The small proportion of injury records that are cause-coded, however, represents a challenge for leaders, policy-makers, safety professionals, researchers, public health scientists, and others interested in data-driven injury prevention, since records do not completely reflect the distributions of mechanisms, activities, and places of occurrence for all injuries, in particular overuse injuries. In addition, use of non-specific or ‘Other, specified’ cause codes is high, offering minimal to no value for prevention, monitoring, and treatment.

Limitations of this analysis included use of the first 9 diagnoses only, although effects should be minimal, since 99.5% of diagnoses are recorded in the first 9 ‘DX’ fields. An additional limitation was the need to exclude data from 4 military treatment facilities that were the first to transition the MHS GENESIS records system. Exclusion was necessary to minimize effects of data missingness during the analysis period.

In summary, results indicate that relatively few military injury electronic medical records, overall, receive a cause code of any kind. Next steps for DOD leaders and policy-makers include efforts to improve cause coding, considering suggestions offered by the Centers for Disease Control and Prevention (CDC),^28^ as well as changes to U.S. military medicine policies, procedures, and contracts to increase injury cause coding. CDC recommendations include integration of cause coding into data standards, development of a toolkit on use of cause codes to set priorities and evaluate injury prevention programs, and creation of guidelines and training to instruct health care providers on injury documentation in medical records.^28^ Providers need support, training, and innovative tools to cause code efficiently and accurately. Ultimately, knowledge of causes is a foundation for the reduction of the burden of injuries on the military medical system and sustainment of military medical readiness.

## Figures and Tables

**Table 1:** Percentage of Incident Injuries^a,b^ with at Least 1 Specified Cause Code Mechanism, Activity, or Place of Occurrence by Service, Active Component, U.S. Armed Forces, 2016–2019

Cause-Coded Injury Encounters	**Total, All Services**		**Army**		**Navy**		**Air Force**		**Marine Corps**	
	No.	%	No.	%	No.	%	No.	%	No.	%
**Any external cause code^c^**										
2016	138,448	9.2	70,183	10.0	22,816	9.6	30,233	8.0	15,216	8.4
2017	147,754	10.0	75,265	11.0	23,826	10.2	32,278	8.5	16,385	9.0
2018	148,990	10.4	75,009	11.7	24,362	10.4	32,927	8.7	16,692	8.9
2019	159,212	10.3	81,310	11.7	25,438	10.0	34,629	8.8	17,835	8.8
2016-2019	594,404	10.0	301,767	11.1	96,442	10.0	130,067	8.5	66,128	8.8
**Mechanism**										
2016	101,281	6.8	50,399	7.2	17,566	7.4	21,932	5.8	11,384	6.3
2017	117,832	8.0	60,450	8.8	20,102	8.6	24,638	6.5	12,642	6.9
2018	122,411	8.5	62,490	9.8	20,769	8.9	25,898	6.8	13,254	7.1
2019	131,105	8.5	67,934	9.8	22,026	8.6	27,294>	6.9	13,851	6.8
2016-2019	472,629	7.9	241,273	8.9	80,463	8.4	99,762	6.5	51,131	6.8
**Activity**										
2016	53,208	3.5	28,654	4.1	7,689	3.2	10,504	2.8	6,361	3.5
2017	57,560	3.9	30,031	4.4	8,354	3.6	11,700	3.1	7,475	4.1
2018	55,844	3.9	28,709	4.5	8,543	3.6	11,574	3.1	7,018	3.8
2019	59,012	3.8	31,051	4.5	8,193	3.2	12,186	3.1	7,582	3.7
2016-2019	225,624	3.8	118,445	4.4	32,779	3.4	45,964	3.0	28,436	3.8
**Place of occurrence**										
2016	35,503	2.4	12,783	1.8	6,852	2.9	10,992	2.9	4,872	2.7
2017	38,078	2.6	15,508	2.3	6,698	2.9	10,708	2.8	5,164	2.8
2018	37,656	2.6	14,509	2.3	7,883	3.4	9,998	2.6	5,266	2.8
2019	38,089	2.5	14,825	2.1	7,664	3.0	9,969	2.5	5,631	2.8
2016-2019	149,326	2.5	57,625	2.1	29,097	3.0	41,667	2.7	20,933	2.8

Abbreviation: No., number.

^a^Excludes encounters that received only cause codes for unspecified mechanisms (T14.91, X58.X, Y09.X, Y35.9, Y36.89, Y36.90, Y37.90, Y38.80), unspecified activity (Y93.3), and/or unspecified place (Y92.9); 2016 n=2,499, 2017 n=1,305, 2018 n=1,251, 2019 n=1,138.

^b^Incident injuries defined by the Taxonomy of Injuries, with a 60-day gap-in-care incidence rule applied to injury type and injured body region. Total incident injury encounters: 2016: 1,500,090; 2017: 1,481,180; 2018: 1,438,012; 2019: 1,554,712.

^c^Note: “Any” category may not equal sum of subcategories, since encounters may have multiple mechanism, activity, and/or place codes.

**Table 2:** Percentage of Incident Injuries with at Least 1 Specified External Cause Code^a,b^ by ICD-10-CM Chapter, Care Source, Clinic Type, Military Hospital or Clinic Size, and Service, Active Component, U.S. Armed Forces, 2016–2019

		**Total, All Services**		**Army**		**Navy**		**Air Force**		**Marine Corps**	
		No.	%	No.	%	No.	%	No.	%	No.	%
**ICD-10-CM chapter**
	**S00-T99**										
	2016	121,916	36.4	60,762	42.1	21,000	33.1	26,349	33.7	13,805	28.3
	2017	130,565	41.4	66,195	48.0	21,858	37.5	27,823	38.1	14,689	32.0
	2018	131,262	42.6	65,931	49.2	22,119	38.8	28,273	39.1	14,939	33.1
	2019	138,852	44.0	70,411	50.7	23,160	39.3	29,544	40.6	15,737	34.7
	**M00-M99**										
	2016	15,609	1.5	8,977	1.7	1,659	1.1	3,645	1.3	1,328	1.1
	2017	16,074	1.5	8,555	1.7	1,797	1.1	4,147	1.4	1,575	1.3
	2018	16,525	1.6	8,462	1.8	1,983	1.2	4,443	1.6	1,637	1.2
	2019	18,933	1.7	10,183	2.0	1,964	1.1	4,823	1.6	1,963	1.3
	**Other**										
	2016	923	1.0	444	1.1	157	1.0	239	1.2	83	0.7
	2017	1,115	1.3	515	1.3	171	1.1	308	1.5	121	1.0
	2018	1,203	1.5	616	1.7	260	1.7	211	1.1	116	1.1
	2019	1,427	1.6	716	1.8	314	1.7	262	1.3	135	1.2
**Care source**
	**Military hospital or clinic (direct care)**										
	2016	121,731	9.5	65,019	10.4	18,639	9.4	24,996	8.2	13,077	8.3
	2017	130,648	10.3	69,810	11.5	19,924	10.3	26,595	8.6	14,319	9.0
	2018	130,461	10.8	69,214	12.3	19,996	10.5	26,691	8.9	14,560	9.0
	2019	139,528	10.7	75,020	12.3	20,789	10.1	28,085	9.1	15,634	8.9
	**Non-military (outsourced care)**										
	2016	16,717	7.7	5,164	6.4	4,177	10.5	5,237	7.3	2,139	8.6
	2017	17,106	8.0	5,455	7.1	3,902	9.7	5,683	7.8	2,066	8.7
	2018	18,529	8.2	5,795	7.4	4,366	9.9	6,236	7.9	2,132	8.8
	2019	19,684	8.0	6,290	7.4	4,649	9.5	6,544	7.7	2,201	8.5
**Visit type**
	**Inpatient**										
	2016	976	32.0	522	35.5	164	29.3	128	22.7	162	35.8
	2017	1,061	34.4	569	38.1	166	29.4	146	29.4	180	38.9
	2018	1,207	39.1	600	42.3	205	36.2	175	29.2	227	44.9
	2019	1,188	40.5	614	44.2	219	40.2	160	28.5	195	44.2
	**Outpatient**										
	2016	137,472	9.2	69,661	9.9	22,652	9.6	30,105	8.0	15,054	8.3
	2017	146,693	9.9	74,696	10.9	23,660	10.1	32,132	8.5	16,205	8.9
	2018	147,783	10.3	74,409	11.7	24,157	10.3	32,752	8.7	16,465	8.8
	2019	158,024	10.2	80,696	11.7	25,219	9.9	34,469	8.8	17,640	8.7
**Size of military hospital or clinic**
	**Hospital**										
	2016	37,097	17.5	17,876	18.8	5,541	18.0	8,820	14.8	4,860	17.9
	2017	38,422	18.6	18,687	20.6	5,143	18.0	8,932	15.0	5,660	20.3
	2018	39,528	19.6	18,882	21.8	4,622	18.0	9,715	15.9	6,309	22.6
	2019	41,564	19.5	20,137	21.7	5,117	16.2	10,141	15.6	6,169	20.0
	**Medical center**										
	2016	33,730	17.0	17,197	16.9	8,388	17.5	4,120	14.9	4,025	19.2
	2017	35,016	17.8	17,011	17.0	9,559	19.5	4,303	15.7	4,143	20.0
	2018	35,717	19.1	17,290	19.1	10,064	21.4	4,096	14.9	4,267	21.4
	2019	37,495	19.5	18,482	19.5	10,207	21.4	4,175	14.9	4,631	21.3
	**Clinic**										
	2016	50,273	6.1	29,683	7.4	4,627	4.0	12,041	5.5	3,922	4.1
	2017	56,528	6.9	33,810	8.7	5,114	4.5	13,341	6.1	4,233	4.3
	2018	54,640	7.0	32,701	9.2	5,369	4.6	12,851	6.1	3,819	2.9
	2019	59,639	7.1	35,908	9.3	5,404	4.3	13,653	6.4	4,674	4.1
**Type of military outpatient clinic**
	**Emergency**										
	2016	61,348	53.1	28,582	55.0	12,742	55.2	11,948	50.8	8,076	47.7
	2017	61,839	57.1	26,732	58.0	13,444	60.4	13,347	56.5	8,316	50.6
	2018	64,213	57.0	27,745	59.6	13,852	60.3	13,773	55.9	8,843	48.0
	2019	68,224	56.4	29,775	60.1	14,587	58.6	14,371	57.3	9,491	44.3
	**Primary care**										
	2016	45,398	6.3	29,061	8.1	3,539	3.8	9,640	4.8	3,158	4.5
	2017	52,687	7.4	34,969	10.2	3,781	4.1	10,100	5.0	3,837	5.0
	2018	50,209	7.4	33,256	10.5	3,252	3.7	9,812	5.1	3,389	5.0
	2019	53,824	7.4	36,614	10.6	3,136	3.1	10,494	5.3	3,580	4.2
	**Specialist**										
	2016	8,747	2.3	3,313	1.8	1,983	2.8	2,082	2.6	1,379	2.6
	2017	10,275	2.6	4,375	2.3	2,271	3.2	1,973	2.5	1,656	3.2
	2018	10,514	2.8	4,717	2.7	2,453	3.6	1,942	2.5	1,402	2.6
	2019	11,831	2.9	4,965	2.7	2,657	3.5	2,019	2.5	2,190	3.7

Abbreviation: ICD-10-CM, International Classification of Diseases, 10th Revision, Clinical Modification.

^a^ Excludes encounters that received only cause codes for unspecified mechanism (T14.91, X58.X, Y09.X, Y35.9, Y36.89, Y36.90, Y37.90, Y38.80), unspecified activity (Y93.3), and/or unspecified place (Y92.9); 2016 n=2,499, 2017 n=1,305, 2018 n=1,251, 2019 n=1,138.

^b^ Incident primary injury diagnoses only. Mechanism, activity, or place of occurrence code.

**Table 3:** Distribution of Specified Mechanisms^a^ for Incident Injuries, Active Component, U.S. Armed Forces, 2016–2019

Mechanism^b^	**2016**		**2017**		**2018**		**2019**	
	No.	%	No.	%	No.	%	No.	%
**Overexertion^c^**	835	0.8	19,846	16.4	23,808	18.9	26,844	19.9
**Falls, slips, trips**	24,034	23.1	22,960	18.9	23,024	18.3	25,244	18.7
**Struck by, against**	22,506	21.6	22,367	18.4	22,460	17.8	23,432	17.4
**Other specified**	16,732	16.1	14,867	12.3	13,829	11.0	14,568	10.8
*Other specified, child or adult abuse*	7,588	7.3	6,666	5.5	5,785	4.6	6,010	4.5
*Other specified, classifiable*	4,848	4.7	4,367	3.6	4,397	3.5	4,821	3.6
*Other specified, foreign body*	3,628	3.5	3,498	2.9	3,276	2.6	3,384	2.5
*Other specified, NEC*	668	0.6	336	0.3	371	0.3	353	0.3
**Motor vehicle traffic (MVT)**	11,832	11.0	11,738	9.7	12,347	9.8	12,928	9.6
*MVT–occupant*	9,555	9.2	9,618	7.9	10,176	8.1	10,719	8.0
*MVT–motorcyclist*	1,850	1.8	1,742	1.4	1,680	1.3	1,691	1.3
*MVT–pedestrian*	199	0.2	169	0.1	237	0.2	268	0.2
*MVT–pedal cyclist*	213	0.2	197	0.2	235	0.2	233	0.2
*MVT–unspecified*	14	0.1	7	<.1	13	<.1	12	<.1
*MVT–other*	1	<.1	5	<.1	6	<.1	5	<.1
**Cut, pierce**	8,565	8.2	9,323	7.3	9,422	7.5	10,064	7.5
**Natural, environmental**	7,822	7.5	8,330	6.9	9,369	7.4	9,548	7.1
*Bites and stings, non-venomous*	3,763	3.6	4,393	3.6	5,157	4.1	5,171	3.8
*Bites and stings, venomous*	2,376	2.3	2,323	1.9	2,335	1.9	2,508	1.9
*Natural, environmental other*	1,683	1.6	1,614	1.3	1,877	1.5	1,869	1.4
P**oisoning**	3,536	3.4	3,839	3.2	3,919	3.1	3,918	2.9
*Poisoning, drug*	2,268	2.2	2,461	2.0	2,690	2.1	2,584	1.9
*Poisoning, non-drug*	1,268	1.2	1,378	1.1	1,229	1.0	1,334	1.0
**Other transport**	2,228	2.1	2,037	1.7	2,062	1.6	2,095	1.6
**Motor vehicle, non-traffic**	1,589	1.5	1,624	1.3	1,632	1.3	1,710	1.3
**Fire, burn**	1,308	1.3	1,332	1.1	1,272	1.0	1,323	1.0
*Hot object, substance*	1,001	1.0	1,026	0.8	997	0.8	1,053	0.8
*Fire, flame*	307	0.3	306	0.3	275	0.2	270	0.2
**Other land transport**	812	0.8	876	0.7	629	0.5	767	0.6
**Pedal cyclist, other**	732	0.7	797	0.7	760	0.6	765	0.6
**Machinery**	927	0.9	622	0.5	566	0.4	627	0.5
**Firearm**	348	0.3	401	0.3	398	0.3	434	0.3
**Pedestrian, other**	180	0.2	212	0.2	165	0.1	189	0.1
**Suffocation**	92	<.1	114	0.1	149	0.1	178	0.1
**Drowning, submersion**	46	<.1	47	<.1	38	<.1	53	<.1
**Total**	104,124	100	121,332	100	125,849	100	134,687	100

Abbreviations: No., number; NEC, not elsewhere classified; MVT, motor vehicle traffic; ICD-10-CM, International Classification of Diseases, 10th Revision, Clinical Modification.

^a^ Excludes cause codes for unspecified mechanisms (T14.91, X58.X, Y09.X, Y35.9, Y36.89, Y36.90, Y37.90, Y38.80); 2016 n=19,236; 2017 n=13,890; 2018 n=13,151; 2019 n=14,386.

^b^ Ordered by 2019 results; main categories bolded, subcategories italicized.

^c^ ICD-10-CM external cause code for “Overexertion from strenuous movement or load,” X50.0, was not available until Oct. 2016.

**Table 4:** Leading Activities^a^ and Places of Occurrence^b^ Associated with Injuries, Active Component, U.S. Armed Forces, 2016–2019

	**2016^d^**		**2017^e^**		**2018^f^**		**2019^g^**	
	No.	%	No.	%	No.	%	No.	%
**Activity^c^**								
Running (Y93.02)	9,985	18.5	11,721	20.2	11,444	20.4	12,556	21.1
Other specified activity (Y93.83)	7,759	14.4	7,442	12.8	7,380	13.1	6,850	11.5
Walking, marching, hiking (Y93.01)	4,136	7.7	5,058	8.7	5,195	9.2	6,298	10.6
Basketball (Y93.67)	4,876	9.2	5,719	9.9	5,191	9.2	5,059	8.5
Free weights (Y93.B3)	2,250	4.2	2,424	4.2	2,326	4.1	2,568	4.3
Other involving muscle-strengthening exercises (Y93.B9)	1,879	3.5	1,872	3.2	1,862	3.3	2,239	3.8
American tackle football (Y93.61)	2,614	4.9	2,398	4.1	2,044	3.6	2,114	3.6
Martial arts (Y93.75)	1,740	3.2	1,845	3.2	1,869	3.3	2,092	3.5
Push-ups, pull-ups, sit-ups (Y93.B2)	1,304	2.4	1,574	2.7	1,521	2.7	1,702	2.9
Soccer (Y93.66)	1,755	3.3	1,778	3.1	1,694	3.0	1,600	2.7
**Place of occurrence^c^**								
Military training ground (Y92.84)	2,812	7.9	4,260	11.2	4,930	13.1	4,972	13.0
Other specified place (Y92.89)	3,559	10.0	3,920	10.3	4,315	11.4	3,493	9.2
Unspecified place in private residence (Y92.009)	3,519	9.9	4,136	10.9	3,724	9.9	3,476	9.1
Other specified sports and athletic area (Y92.39)	3,373	9.5	3,372	8.8	3,061	8.1	3,098	8.1
Unspecified street and highway (Y92.410)	2,576	7.2	2,398	6.3	2,599	6.9	2,635	6.9
Other place on military base (Y92.138)	1,785	5.0	2,099	5.5	1,791	4.8	2,362	6.2
Unspecified place on military base (Y92.139)	1,159	3.3	1,245	3.3	1,242	3.3	1,770	4.6
Other athletic field (Y92.328)	1,427	4.0	1,654	4.3	1,568	4.2	1,742	4.6
Unspecified place in single family (private) house (Y92.019)	1,347	3.8	991	2.6	1,093	2.8	1,287	3.4
Basketball court (Y92.310)	1,444	4.1	1,518	4.0	1,297	3.4	1,231	3.2

Abbreviation: No., number.

^a^Excludes Y93.9, “Activity, unspecified” (2016 n=3,540; 2017 n=2,802; 2018 n=2,729; 2019 n=2,125).

^b^Excludes Y92.9 “Unspecified place or not applicable” (2016 n=9,525; 2017 n=8,027; 2018 n=7,388; 2019 n=7,096).

^c^Ordered by 2019 results; top 10 codes for 2019.

^d^2016, all other specified activities n=15,474 (29% of all specified activities), total specified activities n=53,868; all other specified places n=12,563 (35% of all specified places), total specified places n=35,564.

^e^2017, all other specified activities n=16,104 (28%), total specified activities n=57,935; all other specified places n=12,518 (33%), total specified places n=38,111.

^f^2018, all other specified activities n=15,695 (28%); total specified activities n=56,211; all other specified places n=12,121 (32%), total specified places n=37,687.

^g^2019, all other specified activities n=16,275 (27%), total specified activities n=59,353; all other specified places n=12,059 (32%), total specified places n=38,125.

## AUTHOR AFFILIATIONS

Injury Prevention Branch, Defense Health Agency Public Health–Aberdeen, MD: Dr. Canham-Chervak, Dr. Schuh-Renner, Ms. Rappole; Epidemiology and Analysis Branch, Armed Forces Health Surveillance Division, Public Health Directorate, Defense Health Agency, Silver Spring, MD: Dr. Stahlman; Clinical Public Health and Epidemiology Directorate, Defense Centers for Public Health–Aberdeen: Dr. Jones
